# Root morphometry of the lateral incisor as a contributing factor to the impaction of maxillary canines: A comparative study

**DOI:** 10.4317/jced.63385

**Published:** 2025-12-30

**Authors:** Carla Margarita Castillo-Alcoser, Luis Ernesto Arriola-Guillén, Marjory Elizabeth Vaca-Zapata, Maria Soledad Peña-Herrera, Yalil Augusto Rodríguez-Cárdenas

**Affiliations:** 1 DDS. Orthodontic student, School of Dentistry, Hemisferios University, Quito, Ecuador; 2 DDS, MSc, PhD. Associate Professor of the Division of Orthodontics and Division of Oral and Maxillofacial Radiology, School of Dentistry, Universidad Científica del Sur, Lima, Perú; 3 DDS, MSc, Associate Professor of the Division of Orthodontics, School of Dentistry, Hemisferios University, Quito, Ecuador; 4 DDS, MSc, Associate Professor of the Division of Endodontics and Bucal and Maxillofacial Radiology, School of Dentistry, Hemisferios University, Quito, Ecuador; 5 DDS, MSc, PhD. Assistant Professor of the Division of Oral and Maxillofacial Radiology, Faculty of Dentistry, Universidad Nacional de Colombia, Bogotá D.C, Colombia

## Abstract

**Background:**

This study aimed to evaluate root morphometry of the lateral incisor using cone beam computed tomography (CBCT) as an etiological factor in the occurrence of impacted maxillary canines (IMC).

**Material and Methods:**

A total of 99 CBCT scans from individuals of both sexes were analyzed, revealing 139 impacted maxillary canines, categorized as buccal, palatal, and bicortical, across sagittal and coronal sections of the adjacent incisors (AI). Thus, 59 contralateral incisors from the non-impacted side in unilateral cases were examined. The study evaluated several parameters: sagittal root length of the lateral incisor (SRLI), coronal root length of the lateral incisor (CRLI), sagittal root dilaceration angle (SRD), coronal root dilaceration angle (CRD), sagittal root convergence angle (SRC), and coronal root convergence angle (CRC). In total, 198 lateral incisor roots were assessed. The statistical analyses included Chi-square tests, Student's t-tests, and Tukey's tests, with a significance level set at P&lt;0.05.

**Results:**

The study found that cases of canine impaction were more prevalent in females, with the majority being unilateral (47.5%) and located in a palatal position. Bilateral cases were predominantly buccal (52.5%) (p=0.001). Root dimensions on the impacted side were significantly smaller in the sagittal section (14.75 mm) compared to the non-impacted side (15.67 mm) (p=0.001). In the coronal section, measurements were also smaller on the impacted side (mean difference of 0.57 mm), but this difference was not statistically significant (p=0.082). The root lengths in both coronal and sagittal sections were shortest in the bicortical group (12.67 mm and 12.95 mm, respectively) compared to the palatal (15.34 mm coronal / 15.62 mm sagittal) and buccal (14.89 mm coronal / 15.54 mm sagittal) groups (p&lt;0.05).

**Conclusions:**

Individuals with impacted maxillary canines (IMC) exhibit shorter root lengths of the adjacent lateral incisor compared to the non-impacted side, with bicortical IMC cases showing the shortest lengths, approximately 2 mm shorter than other types of impactions. Orthodontists should consider this condition when planning treatments involving IMC.

## Introduction

Dental impaction is defined as the condition in which a tooth remains in an intraosseous position even after its root formation has been completed, either due to the presence of a physical barrier or due to a lack of space for eruption, preventing it from reaching the dental arch within the normal development time (1 , 2). In terms of prevalence, maxillary canines are the second most impacted teeth after mandibular third molars, and they act as guides for occlusal disocclusion, shaping the arch and being a fundamental part of smile aesthetics (3). Becker et al. (4) reported a higher prevalence of maxillary canine impaction in the palatal position in individuals with agenesis of the lateral incisor, conical incisors, or microdontia. Due to various conditions, the prevalence of unilateral canine impaction is higher than bilateral impaction (3 , 5). Among the main theories explaining the etiology of canine impaction is the eruption guidance theory, (6) which states that canines use the distal surface of the lateral incisor root as a guide to reach their position in the arch; if the lateral incisor has any anomaly or is absent, the eruption guidance is lost, potentially causing palatal impaction of the canine (2 , 5). Becker et al. (7) also proposed that canine impaction has no genetic association but occurs as a result of local obstacles. Sacerdotti and Baccetti (8) confirmed a strong relationship between lateral incisor agenesis and unilateral palatal canine impaction, which supports the eruption guidance theory by highlighting the etiological relevance of the adjacent incisor root to the impacted maxillary canine. Garib et al. (9) stated that patients in the mixed dentition phase with a diagnosis of dental anomalies, such as microdontia, are at a higher risk of canine displacement toward the palatal position. It is well-established that three-dimensional images, such as Cone Beam Computed Tomography (CBCT), are radiologically the gold standard in the identification of impacted canines, as they allow for the analysis of their position in all three spatial planes, facilitating the evaluation of the root morphology of the lateral incisor or its location, whether buccal, bicortical, or palatal (10). Several studies on CBCT have reported a significant relationship between the root length of the lateral incisor and the impaction of maxillary canines, specifically finding that it is shorter on the impacted side of buccal and palatal canines compared to the non-impacted side (11). However, to date, these characteristics have not been evaluated or compared, including samples with bicortical IMC, nor have other morphometric root characteristics of the maxillary lateral incisor been assessed. Therefore, identifying root anatomical factors of a lateral incisor that may promote the impaction of a maxillary canine, whether buccal, palatal, or bicortical, would further help define the theory of its etiology. To the authors' knowledge, literature on establishing such indicators for all three types of maxillary canine impaction is scarce. For this reason, the aim of the present study was to compare the root morphology of the lateral incisor as a possible etiological factor in canine impaction, whether buccal, bicortical, or palatal, versus its counterparts on the non-impacted side. The null hypothesis proposed in this study is that there are no differences in the root morphology of the lateral incisor adjacent to a buccal, palatal, or bicortical IMC, nor with that of the contralateral non-impacted tooth.

## Material and Methods

**Figure 1:**
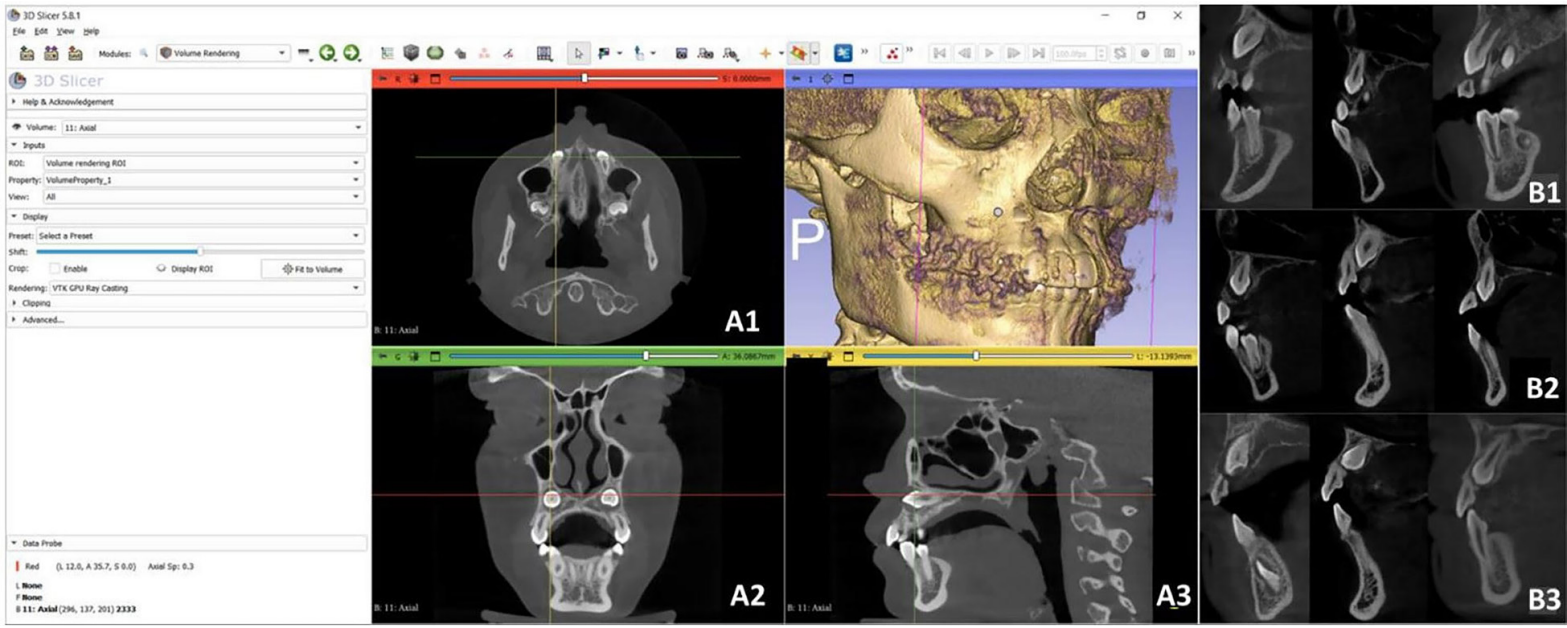
A) Localization of the canine in the 3D Slicer Program version 5.8.1. (A1) Axial Section; (B1) Coronal Section; (C1) Sagittal Section. B) Position of the impacted canine in the sagittal section, showing (B1) Buccal, (B2) Palatal, and (B3) Bicortical views.

This is an observational, descriptive, and cross-sectional study. The sample consisted of 99 CBCT scans of patients with impacted maxillary canines (59 unilateral and 40 bilateral); the sex distribution was 63 females and 36 males, with an average age of 19 years. Prior to its execution, approval was obtained from the Ethics Committee for the approval of thesis proposals at the University of the Hemispheres, Quito, Ecuador, under No. CEUHE25-50. The CBCT scans were performed with a Planmeca ProMax 3D Mid tomograph at 120kV, with FOVs of 20x10mm and 20x17mm, a voxel size of 75 m, and an exposure time of 15 seconds. These tomograms belong to the Tomography Bank of the Orthodontics postgraduate program at Universidad de Los Hemisferios, located in Quito, Ecuador, and are accompanied by a donation letter issued by the University. CBCT scans of subjects aged 12 years and older were included, with impacted maxillary canines (IMC) in buccal, bicortical, and palatal positions; both unilateral and bilateral. The study also considered maxillary lateral incisors with complete apical closure and with or without root resorption. Exclusion criteria included images of patients undergoing or having previously undergone orthodontic treatment, with a history of trauma or maxillofacial surgery, agenesis of lateral incisors, syndromic conditions, or endodontic treatments in the upper lateral incisors, or with root hypoplasia or root hyperplasia. In the 99 individuals analyzed, 139 IMC were observed: The study group consisted of 139 lateral incisors adjacent (LIA) to an IMC. In cases of unilateral impaction, the contralateral lateral incisors on the non-impacted side was used for the control group, which included 59 incisors. Each lateral incisor was considered a sampling unit for analysis. The DICOM files were imported into the 3D Slicer 5.8.1 software to determine the localization of IMC as buccal, palatal, or bicortical. The coronal and sagittal sections were aligned with the long axis of the tooth, and in the axial section, the incisal edge was aligned with the coronal plane. Linear and angular measurements of the maxillary lateral incisors were performed in the sagittal and coronal sections (Fig. 1).

1

**Figure 2:**
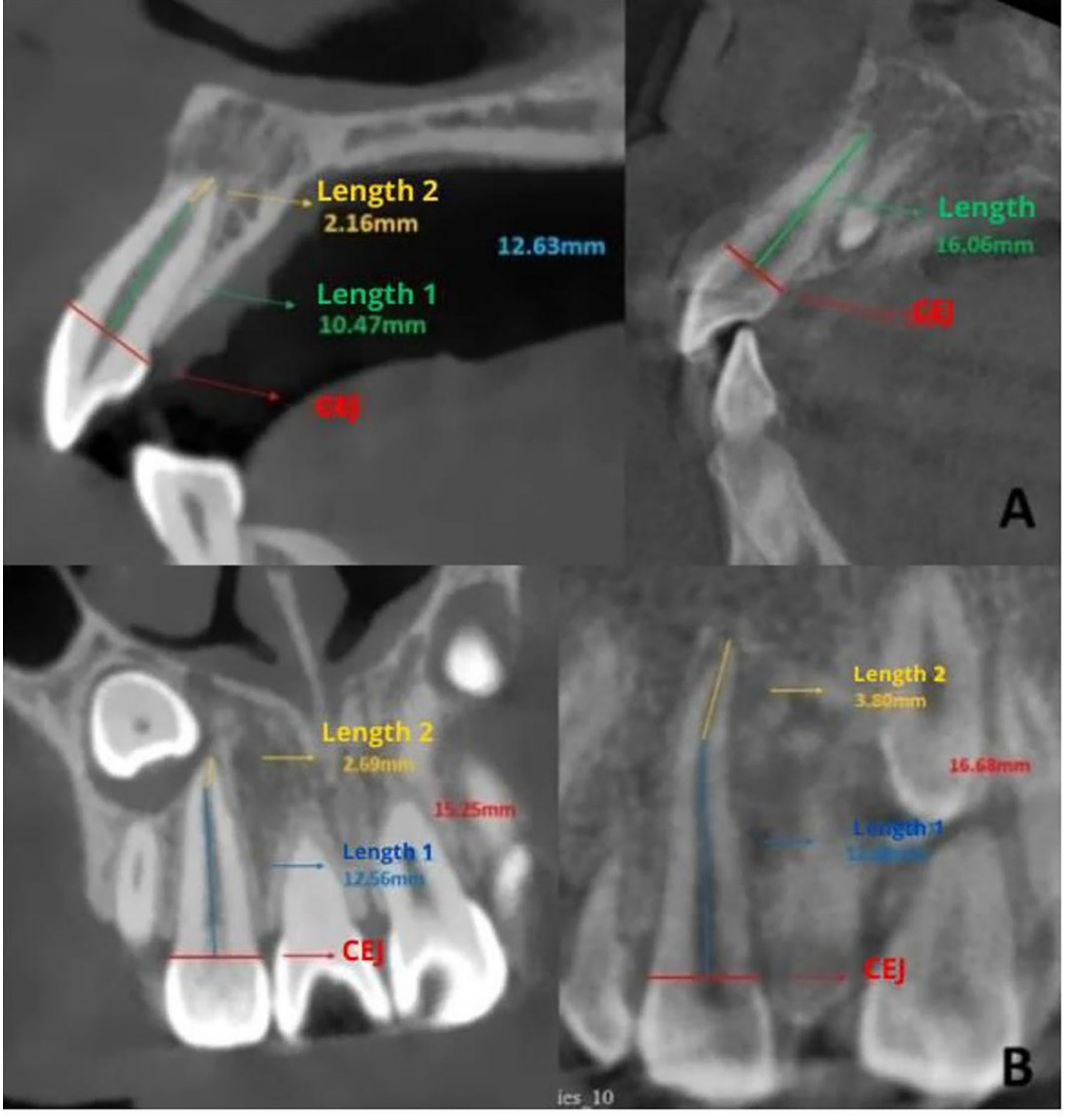
A) Sagittal Root Length of the Lateral Incisor (SRLI); B) Coronal Root Length of the Lateral Incisor (CRLI).

**Figure 3:**
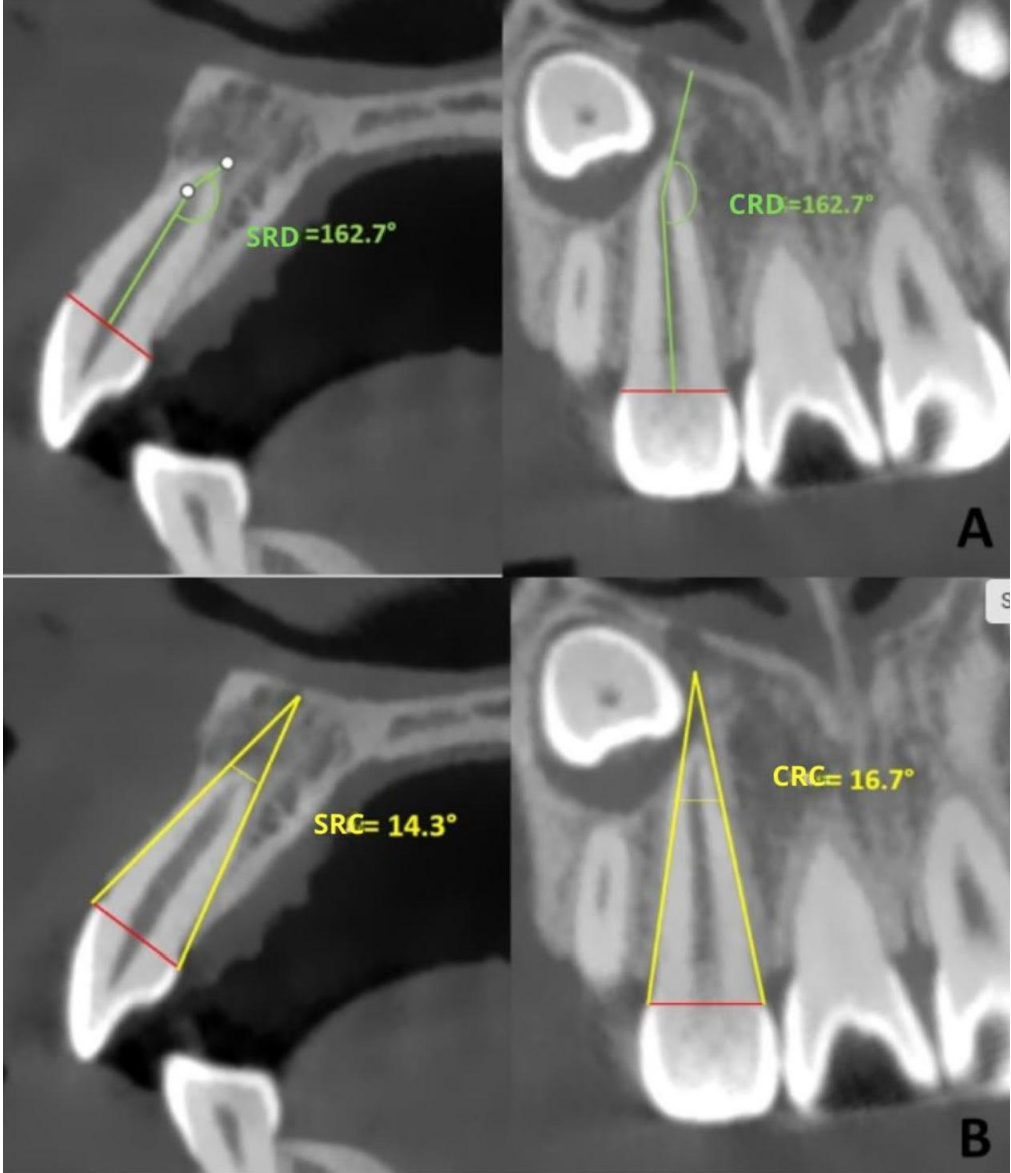
A) Sagittal Root Dilaceration Angle (SRD) and Coronal Root Dilaceration Angle (CRD); B) Sagittal Root Convergence Angle (SRC) and Coronal Root Convergence Angle (CRC).

Additionally, the following measurements were taken on both the control and study groups: sagittal root length of the lateral incisor (SRLI), coronal root length of the lateral incisor (CRLI) (Fig. 2), sagittal root dilaceration angle (SRD) and coronal root dilaceration angle (CRD); and sagittal root convergence angle (SRC) and coronal root convergence angle (CRC) (Fig. 3).

2

3

For the root measurements of the lateral incisor, a line was drawn from mesial to distal and from buccal to palatal at the cementoenamel junction level in the sagittal and coronal sections of the tooth. Along this line, and from the center of the root canal, another line was drawn to the start of the root dilaceration; and from there, another line was drawn to the apex. The sum of these two measurements constituted the sagittal root length (SRLI) and coronal (CRLI). Additionally, the starting point of the dilaceration served as the vertex for measuring the sagittal dilaceration angle (SRD) and coronal (CRD) of the lateral incisor. The sagittal convergence angle (SRC) and coronal (CRC) were obtained using reference points at the cementoenamel junction, drawing two tangents that followed the root contour in the sagittal and coronal sections. The point where they intersected was defined as the vertex, using the same measurement performed in a previous study (3). An orthodontist with 10 years of experience in the informed reading of IMC acted as the gold standard for the training of the main evaluator. The measurements were taken twice by the same evaluator, with a 15-day interval. The weighted intraclass correlation coefficients for intra-examiner measurement error, calculated using STATA version 16, showed values greater than 0.90 for all measurements, interpreted according to the Landis and Koch scale (12). - Statistical Analysis All data were recorded in an Excel spreadsheet and subsequently analyzed using the SPSS statistical package version 19.0 (IBM SPSS, Chicago, Illinois, USA). The normality of the data was verified using the Shapiro-Wilk test. Intergroup comparisons were performed using the Student's t-test, and associations were evaluated using the chi-square test. Statistical significance was set at 0.05 for all tests.

## Results

Table 1 describes the initial characteristics of the sample according to sex and age.

Table 1

Table 2 shows the distribution of the sample by side of impaction and type of impaction, finding that most of the unilateral cases were palatal (47.5%), while the bilateral cases were buccal (52.5%) (p=0.001).

Table 2

Table 3 summarizes the comparisons between groups of lateral incisor root length and the dilaceration and convergence angles.

Table 3

These three measurements were evaluated in both sagittal and coronal sections. The results showed that the root dimensions were smaller in the root of the adjacent incisor (AI) (14.75mm) compared to the dimensions of the same tooth on the non-impacted side (15.67mm) evaluated in the sagittal section, with these differences being statistically significant (p=0.001). Although the measurements were also smaller in the coronal section (mean difference 0.57), no significant differences were found between groups (p=0.082). Table 4 summarizes the root dimensions of the LIA according to the location of the maxillary canine impaction, finding primarily smaller root length values in the sagittal section in the bicortical group (12.95mm) compared to the palatal (15.62mm) or buccal (15.54mm) groups (p&lt;0.05).

Table 4

Similarly, smaller root length values were found in the coronal section in the bicortical group (12.67mm) compared to the palatal (15.34mm) or buccal (14.89mm) groups (p&lt;0.05).

## Discussion

The aim of this study was to compare the root morphometry of the lateral incisor as a possible etiological factor for the impaction of a canine, whether buccal, bicortical, or palatal, versus its counterpart on the non-impacted side. The morphological identification of potential anatomical root factors of the adjacent maxillary lateral incisors (MLI) to IMC could help establish the influence of local factors as a possible cause of impaction and would provide further relevance to the eruption guidance theory, which promotes a predominant role of the root of the MLI in this clinical scenario. In the present study, a higher prevalence of canine impaction was observed in females (64%), data consistent with other studies where the prevalence by sex was 72.3%2, 53.5% (13), and 63% (5). Bilateral impaction was more common, with 57.5%, compared to unilateral impaction (42.5%), and most of the IMC were found in the buccal position (38.8%), followed by palatal and bicortical positions. Discrepant findings were reported by Hossein Razeghinejad et al. (2), in which palatal impaction predominated at 89.4%, compared to buccal impaction at 10.6%; however, this study did not include canines in bicortical position. Root lengths in the sagittal section of the adjacent incisor (AI) to impacted canines compared to adjacent incisors to normally positioned canines showed significant differences between groups, with a mean dimension of 14.75 mm for the impacted side and 15.67 mm for the non-impacted side. These data are consistent with the study by Melchor-Soto et al. (3) where the root length of the LAI showed a tendency to be shorter. Although the difference between groups in that study was minimal, it was concluded that maxillary canine impaction is associated with a shorter root of the LAI. The most important finding of this study was the significant differences found in the root lengths in the sagittal and coronal sections in the intragroup comparison, that is, between the different locations of maxillary canine impaction, particularly the difference found in the LAI to bicortical IMC compared to buccal and palatal impactions. Our data showed that a short root is a risk factor for maxillary canine impaction. This finding is crucial in the initial imaging evaluation recommended for a growing patient, when the orthodontist identifies other clinical risk factors that may favor the presence of this condition, such as the absence of palpation of the canine crown during eruption, asymmetric eruption (14), reduced arch dimensions (15), microdontic or atypical lateral incisors (16), or atypical eruption patterns of the lateral incisor, among others. It could be thought that the root lengths of the LAI are shorter than those of the contralateral incisor due to the proximity and the condition of maxillary canine impaction, and that it could possibly be the result of resorption caused by this tooth. However, it is important to emphasize that in the sample selection, cases where the apex of the LAI was not clearly visible on tomography were excluded. In these cases, its absence was determined to be caused by the maxillary canine impaction, according to the literature and scientific evidence that has established this causality in multiple studies and systematic reviews with meta-analyses (18 - 21). Therefore, early imaging identification of the risk of bicortical impaction of a maxillary canine is essential, mainly because it has been reported that this condition is the most aggressive in terms of root involvement of the lateral incisor, for which severe root resorptions have been reported (22 - 25), and even premature loss, especially in growing patients. This aspect reinforces the clinical applicability of the findings in this study. At this point, it is important to highlight that the eruption of a maxillary canine or any tooth is a multifactorial and three-dimensional phenomenon, influenced by various aspects that interrelate individually to a greater or lesser extent in each case, such as the position of the dental germ, available space, genetic and environmental factors, among others. In some of these factors, scientific evidence is still insufficient, or the technology to evaluate them is not yet accessible to clinicians (26). Our results support the eruption guidance theory, and additionally, suggest that other morphological traits of the lateral incisor root, the maxillary bone, or the canine germ, may also be determinants in the etiology and/or location of a maxillary canine impaction (MCI). Regarding the comparison of the angulation of root dilaceration between groups, no significant differences were found. The average was 157.31° for the impacted side and 155.52° for the unaffected side, the sagittal (SRD) and coronal (CRD). This angle describes the level of mesial and distal inclination of the apical root portion of the adjacent incisor. It could be thought to play a predominant role in impaction as a potential etiological factor: the more closed the dilaceration angle, the greater the likelihood of impaction, as it could act as a mechanical obstacle to the eruption guidance of the canine. However, the fact that no significant differences were found between groups in the evaluation of the lateral incisor root in the present study does not necessarily mean that other unexamined morphometric traits could not be associated. Similarly, the root convergence angle was similar in both groups, which is an indicator of the sagittal and coronal root volume of the incisor. This aspect is also important as an etiological factor, given that certain developmental anomalies in maxillary incisors have a significant association with canine impaction (5). It is important to note that the comparison was made with incisors from the non-impacted side of unilateral impaction cases. It is possible that the same results may not be seen in comparison with a group without any impaction. Further studies with larger sample sizes are needed to determine whether these associations exist or not. Finally, the null hypothesis of the study is partially accepted, as only significant differences were found in the sagittal root length of the LIA compared to the contralateral unaffected incisor. The limitations of this study were primarily represented by the sample size, as well as data such as demographic or ethnic origin, which could be etiological characteristics of canine impaction, related to genetic theory. The number of sample units in the study group was 139 MLI, and in the control group, it was 61 MLI. This difference in sample sizes between groups could have been a bias factor that influenced the results. We recommend future studies with a larger sample size of CBCTs and with information from patients sharing certain characteristics that allow for evaluating these comparisons between matched groups.

## Conclusions

Individuals with IMC exhibit shorter root lengths of the adjacent lateral incisor compared to their counterparts on the unaffected side, with the shortest root lengths observed in bicortical IMC cases, approximately 2 mm shorter than other types of impaction. Orthodontists should consider this condition when planning treatments involving impacted maxillary canines.

## Figures and Tables

**Table 1:** Initial sample characteristics (n=99).

Sex	n	Age	
		Mean	SD
Male	36	18.08	7.05
Female	63	19.27	8.23

p=0.470, Student’s t-test

**Table 2:** Sample characteristics based on sides affected and impaction location.

Sides affected		Location			
		Buccal	Palatal	Bicortical	Total
Unilateral canines	n	12	28	19	59
	%	20.3	47.5	32.2	100
Bilateral canines	n	42	20	18	80
	%	52.5	25	22.5	100
Total	n	54	48	37	139
	%	38.8	34.5	26.6	100

p=0.001, Chi-square test

**Table 3:** Comparison of upper lateral incisor measurements evaluated between the impacted side and control group.

Measurement	n	Mean	SD	Mean Difference	95% Confidence Interval		p- value
					Lower limit	Upper limit	
Root length (sagittal) impacted side	139	14.75	3.14	-0.91	-1.64	-0.19	0.015*
Root length (sagittal) unaffected side	59	15.67	1.76				
Dilaceration angle (sagittal) impacted side	139	157.31	23.07	1.79	-4.51	8.09	0.571
Dilaceration angle (sagittal) unaffected side	59	155.52	12.57				
Convergence angle (sagittal) impacted side	139	13.34	2.48	0.07	-0.62	0.76	0.839
Convergence angle (sagittal) unaffected side	59	13.27	2.35				
Root length (coronal) impacted side	139	14.65	2.83	-0.57	-1.22	0.08	0.082
Root length (coronal) unaffected side	59	15.22	1.86				
Dilaceration angle (coronal) impacted side	139	161.60	11.58	1.26	-2.72	5.24	0.528
Dilaceration angle (coronal) unaffected side	59	160.34	13.33				
Convergence angle (coronal)impacted side	139	14.75	2.82	0.44	-0.45	1.34	0.326
Convergence angle (coronal) unaffected side	59	14.31	3.07				

*Significant, paired Student’s t-test

**Table 4:** Root dimensions of the upper lateral incisor evaluated based on the location of maxillary canine impaction.

Measurement	Location	n	Mean	SD
Root length (sagittal)	Bucal	54	15.54ª	1.54
	Palatal	48	15.62ª	2.04
	Bicortical	37	12.95b	3.63
Dilaceration angle (sagittal)	Buccal	54	156.17ª	12.10
	Palatal	48	157.93ª	12.08
	Bicortical	32	160.47ª	28.52
Convergence angle (sagittal)	Buccal	54	13.97ª	2.43
	Palatal	48	12.89ª	2.67
	Bicortical	36	13.88ª	2.50
Root length (coronal)	Buccal	54	14.89ª	1.79
	Palatal	48	15.34ª	1.86
	Bicortical	37	12.67b	3.96
Dilaceration angle (coronal)	Buccal	54	160.54ª	11.55
	Palatal	48	162.91ª	12.55
	Bicortical	32	164.68ª	11.79
Convergence angle (coronal)	Buccal	52	14.26ª	2.42
	Palatal	47	14.85ª	2.76
	Bicortical	37	14.86ª	2.76

Different letters indicate significant difference (p< 0.05) according to Tukey’s test.

## Data Availability

The datasets used and/or analyzed during the current study are available from the corresponding author.
